# Embolization of De Novo Pulmonary Arteriovenous Malformations Using High-Volume Detachable Non-Fibered Coils: Propensity-Matched Comparison to Traditional Coils

**DOI:** 10.3390/jcm13030648

**Published:** 2024-01-23

**Authors:** Sipan Mathevosian, Hiro D. Sparks, Lucas R. Cusumano, Dustin G. Roberts, Shamaita Majumdar, Justin P. McWilliams

**Affiliations:** Division of Interventional Radiology, Department of Radiology, David Geffen School of Medicine at UCLA, Los Angeles, CA 90095, USA; smathevosian@mednet.ucla.edu (S.M.);

**Keywords:** Pulmonary Arteriovenous Malformation (PAVM), embolization, Hereditary Hemorrhagic Telangiectasia (HHT), vascular medicine, pulmonary angiography, pulmonary embolization

## Abstract

Embolization of de novo pulmonary arteriovenous malformations (PAVMs) using high-volume detachable non-fibered (HVDNF) coils was compared to traditional non-HVDNF coils. Persistent-occlusion rates were evaluated. A total of 272 de novo (previously untreated) PAVM treatments were retrospectively stratified into those treated with non-HVDNF coils only (*n* = 192) and those treated with HVDNF coils with or without other coils (*n* = 80). Propensity score matching, followed by survival analysis and cost analysis, was performed. The overall persistent-occlusion rate was 86.0% (234/272). Persistent occlusion was achieved in 81.8% of PAVMs using non-HVDNF coils, compared with 96.3% using HVDNF coils (*p* = 0.0017). The mean follow-up was 30.7 ± 31.9 months versus 14.7 ± 13.4 months, respectively (*p* < 0.0001). Propensity-matched survival analysis demonstrated PAVMs treated with HVDNF coils recurred significantly less frequently than PAVMs treated with non-HVNDF coils (*p* = 0.023). The use of HVDNF coils was more expensive than standard coils, however not significantly different for the treatment of complex PAVMs. The use of high-volume detachable non-fibered coils was associated with higher persistent-occlusion rates when compared with non-HVDNF coils. HVDNF coils were more expensive on average; however, cost was similar between groups for the treatment of complex PAVMs.

## 1. Introduction

Pulmonary arteriovenous malformations (PAVMs) are abnormal pulmonary vascular connections that bypass the normal capillary bed and create a right-to-left shunt. Resultant shunting can cause derangements in pulmonary gas exchange, leading to dyspnea and hypoxemia, and reduce capillary filtration, leading to paradoxical embolic stroke or cerebral abscess [1,2,3]. Inherent PAVM fragility can also lead to hemoptysis or hemorrhage [2]. PAVMs are frequently encountered in patients with hereditary hemorrhagic telangiectasia (HHT), an autosomal-dominant genetic vascular disease characterized by epistaxis, mucocutaneous telangiectasias, and visceral AVMs. Approximately 70% or more of PAVMs occur in patients with HHT, and approximately 15 to 50% of patients with HHT have a PAVM, whereas sporadic cases are comparatively uncommon [1,4,5,6]. 

Standard of care treatment for PAVMs is transcatheter embolization with the goal of therapy being durable complete occlusion [3,7,8,9]. Embolization can be performed with a variety of coil or plug embolics. Persistent-occlusion rates after coil embolization of previously untreated PAVMs vary from 51 to >95%, a wide range secondary to different embolic materials, embolic techniques, variability in timing and modality of follow-up, and definitions of persistence [1,3,4,7,8,10,11,12,13,14,15,16,17,18,19,20]. Achieving durable occlusion of de novo PAVMs is important, as treatment of recurrent PAVMs is technically challenging with lower success rates [15,16,21]. 

PAVM embolization was historically performed with detachable balloons or fibered, pushable coils. These technologies gave way to detachable fibered microcoils and a variety of plug embolics, which have shown superior persistent-occlusion rates compared with coils [17,19,22,23,24]. Recently, a high-volume-detachable-non-fibered (HVDNF)-coil embolic (Penumbra Inc., Alameda, CA, USA) has gained popularity. HVDNF coils are non-fibered and engineered to maximize volume and packing density, which has been previously shown to predict persistent occlusion of PAVMs [11,15,25,26,27]. The focus of this investigation was to review a single institutional experience of de novo PAVM embolization using high-volume detachable non-fibered coils and compare persistent-occlusion rates and cost to traditional, non-HVDNF coils.

## 2. Materials and Methods

After Institutional Review Board approval (IRB #17-001205) and in accordance with the Health Insurance Portability and Accountability Act, a single-center retrospective study was conducted on patients who underwent embolization of previously untreated PAVMs between February 2008 and October 2019. A total of 272 de novo PAVMs in 108 individual patients were included for review. Excluded from analysis were previously embolized PAVMs requiring retreatment, as well as truly diffuse PAVMs defined as PAVMs involving every subsegmental branch of at least one lung segment. Persistent-occlusion rates with different embolic materials were compared, with specific attention to the use of high-volume detachable non-fibered (HVDNF) coils (Penumbra Inc., Alameda, CA, USA). Persistent occlusion on follow-up CT angiography (CTA) of the chest was defined as more than a 70% decrease in the PAVM sac or draining vein. Persistent flow through the embolic (recanalization) or around the embolic (reperfusion) defined treatment failure.

### 2.1. Treatment Groups

PAVMs (*n* = 272) were retrospectively stratified into those treated with traditional, non-HVDNF coils only (*n* = 192) and those treated with HVDNF coils with or without other coils (*n* = 80). Persistent occlusion was the primary endpoint. 

### 2.2. Additional Variables

Baseline variables, including age, sex, and HHT diagnosis, were recorded. The type and number of coils used in each PAVM were obtained from the operative note, implantable device records, and review of the angiographic images. The coil length and diameters for HVDNF coils were also recorded. Feeding-artery diameters were obtained from the operative note and calculated by pixel-ratio estimation comparing to the base catheter on angiographic images.

PAVM angioarchitecture was defined as simple or complex at the time of pulmonary angiography, based on conventional nomenclature [28]. Simple PAVMs were defined as having a single feeding pulmonary artery, whereas complex PAVMs had two or more feeding pulmonary arteries. 

Additionally, a cost analysis was performed, where the individual price of each coil embolic, based on institutional purchase price, was used to calculate the overall cost of each of the 272 individual PAVM treatments. 

### 2.3. Technique

The majority of embolizations were performed by a single operator with 1–10 years of experience over the course of the study (93.4%, *n* = 254); the remainder were performed by an additional 5 operators with 7–42 years of experience (6.6%, *n* = 18). Pre-procedural CT angiography of the chest was reviewed for treatment planning. 

Embolization procedures were performed under conscious sedation on an outpatient basis via right common femoral venous access with placement of a long 6F sheath or 7F guide catheter into the main pulmonary artery. Diagnostic pulmonary angiography of the target lung was performed through a 5F pigtail catheter in contralateral oblique projection to evaluate the size and configuration of PAVMs. Selection of the supplying artery to the PAVM was achieved using a 5F hydrophilic catheter or other 5F angiographic catheter. Superselection of the distal feeding artery to the PAVM or the PAVM nidus was achieved with the angiographic catheter or with a variety of 2.4 F to 2.8 F microcatheters.

**Figure 1:**
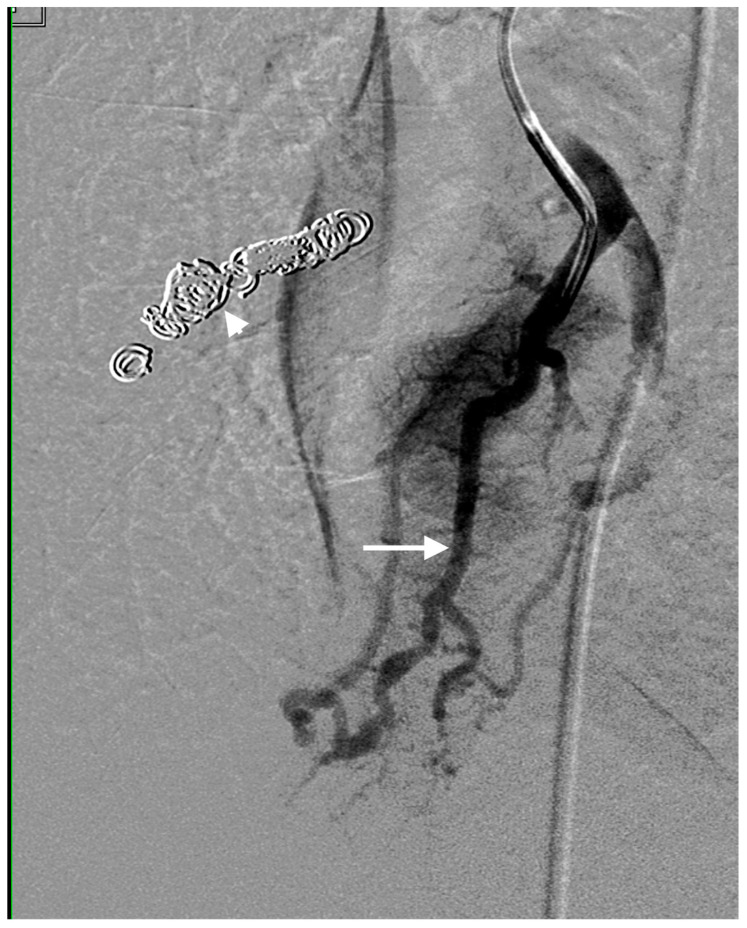
Right-lower-lobe simple PAVM with single feeding artery (straight arrow) and draining veins. Coil pack from a prior embolization is also noted (arrowhead).

**Figure 2:**
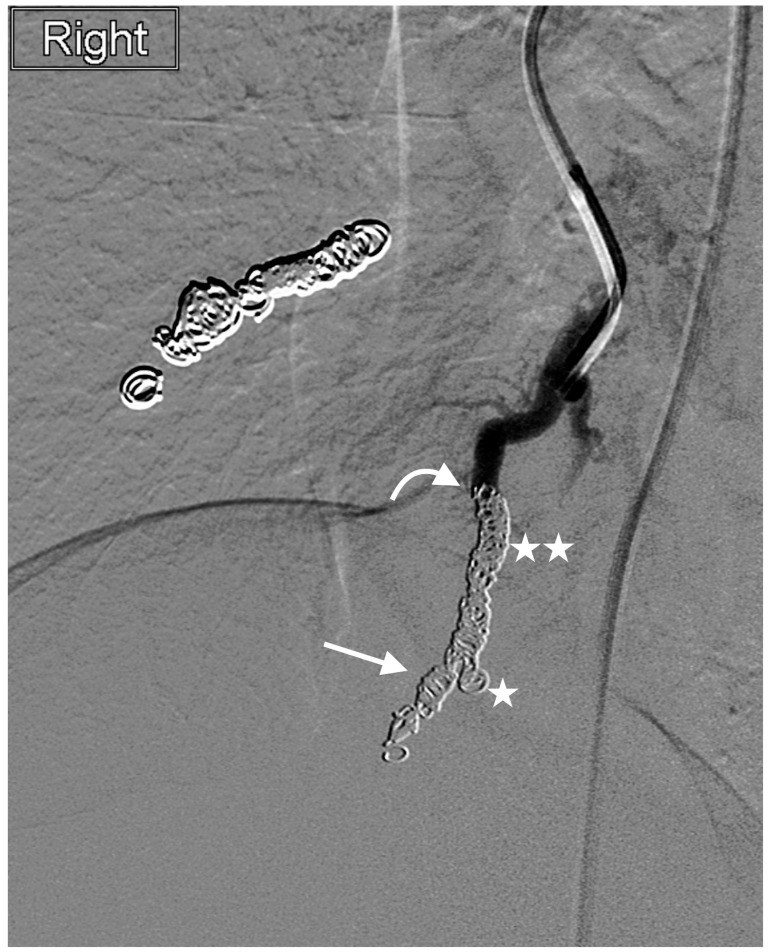
Right-lower-lobe simple PAVM treated with embolization of the nidus (straight arrow) and distal feeding artery (curved arrow) using 3 mm × 12 cm Interlock coils (×5) (star) and 2 mm × 6 cm Interlock coils (×2) (double star) (non-HVDNF coils) with complete occlusion. Note the relative density of the coil pack.

**Figure 3:**
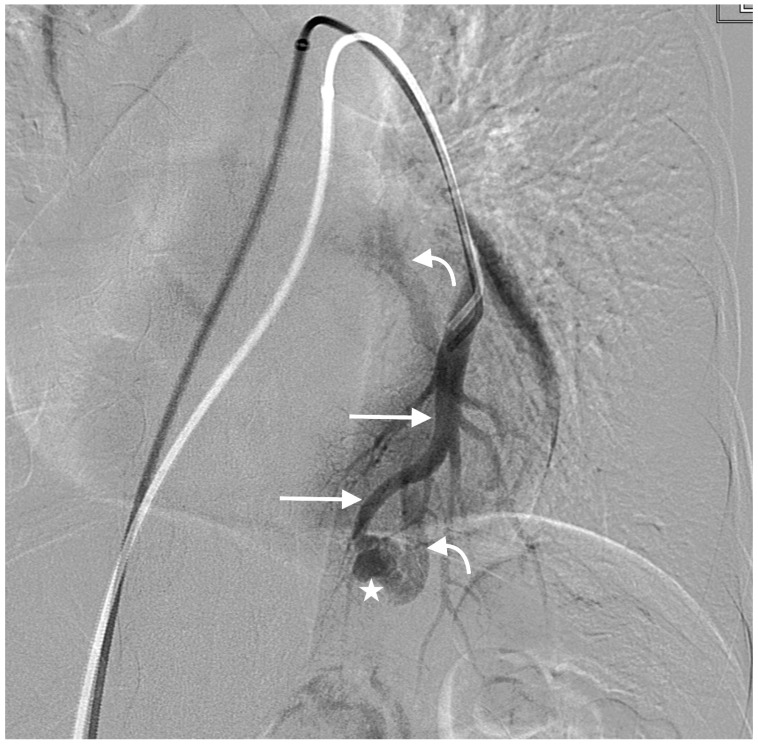
Left-lower-lobe simple PAVM with single feeding artery (straight arrows), sac (star), and draining vein (curved arrows).

**Figure 4:**
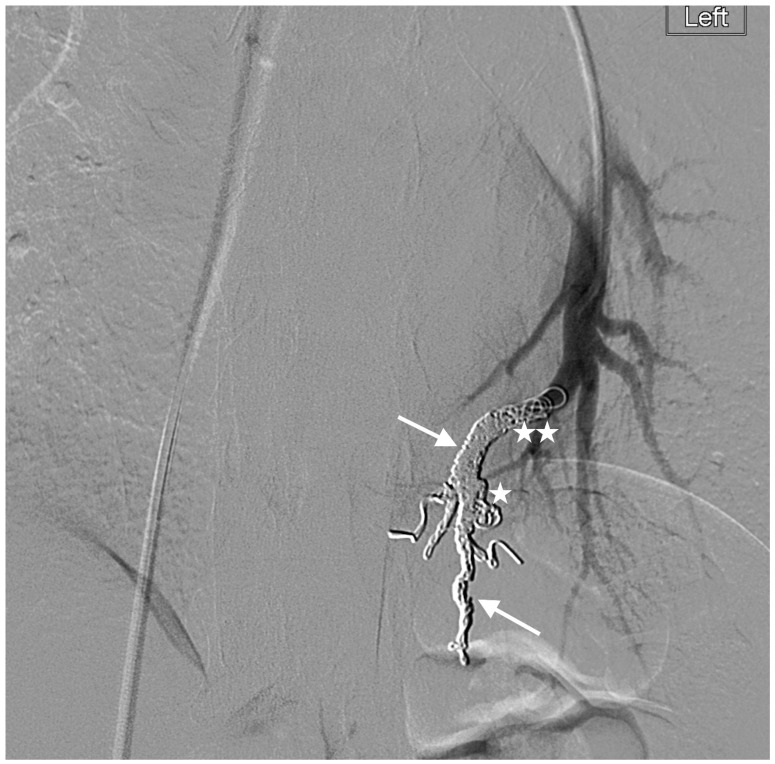
Left-lower-lobe simple PAVM treated with embolization of the nidus (star) and distal feeding artery (double star) using 60 cm Packing Coils (×2) (straight arrows) (HVDNF coils) with complete occlusion. Note the relative density of the coil pack.

Embolotherapy was performed with a variety of coils determined at the time of angiography by the performing interventional physician. Coil embolics are included in Table 1. In the first 8 years of the study, embolization was performed primarily with detachable fibered microcoils (Interlock and Interlock Soft, Boston Scientific, Marlborough, MA, USA), sometimes supplemented with pushable fibered coils or microcoils (Nester, Tornado and microNester, Cook, Bloomington, IN, USA) (*n* = 144), and a small number of PAVMs were embolized with detachable low-volume coils (Axium and Concerto, Medtronic, Minneapolis, MN, USA) or detachable hydrogel-coated coils (Azur, Terumo, Somerset, NJ, USA) (*n* = 48) (Figure 1 and Figure 2). In the last 3.5 years, embolization was performed primarily with HVDNF coils (Ruby, Penumbra, Alameda, CA, USA) (*n* = 43) (Figure 3 and Figure 4). In an effort to reduce costs, some PAVMs were embolized with a combination of HVDNF coils, followed by pushable or detachable fibered coils or microcoils (*n* = 37). Embolization was targeted to the distal feeding artery (DFA) (within 1 cm of the nidus), without or with embolization of the nidus (NFA). In rare cases where distal embolization was not possible, embolization of the proximal feeding artery (PFA), more than 1 cm from the nidus, was performed [15,21,29]. 

Patients were seen in clinic with CTA of the chest 3–6 months after embolization to confirm occlusion and then followed with repeat CTA every 3–5 years [21]. The timing of the last clinical follow-up or PAVM recurrence was recorded. 

### 2.4. Statistical Analysis

Continuous data are expressed as mean ± standard deviation with range. Univariate analysis was performed with the chi-square test for categorical variables and the *t*-test, Mann–Whitney test, or Wilcoxon signed-rank test for continuous variables. Univariate statistical analysis was performed using GraphPad PRISM 8.0 (GraphPad Software, San Diego, CA, USA). 

Propensity score matching was performed to compare the treatment effects of HVDNF relative to non-HVDNF coils while minimizing bias related to confounding variables and statistical-modeling-linearity assumptions [30]. Prior to the propensity-matching procedure, covariates that were to be matched were carefully selected to minimize treatment influences not related to coil type and to maximize generalizability. These covariates included age, presence of definitive HHT diagnosis, PAVM feeding-artery diameter, PAVM angioarchitecture (i.e., complex versus simple), embolization technique (i.e., DFA vs NFA), and follow-up time. Prior to matching, it was noted that HVDNF-treated PAVMs were exclusively embolized at the DFA or NFA. Given inferiority of PFA embolization for achieving durable occlusion, the non-HVDNF PAVMs treated at the PFA (*n* = 7) were manually removed from the pool of potential match controls [21,31]. A propensity score was generated for each HVDNF-treated PAVM using a logistic regression of the treatment on the covariates and matched to a non-HVDNF-treated PAVM using the ‘optimal’ algorithm in the ‘MatchIt’ package in R [32]. After matching, all standardized mean differences for the covariates were below 0.15, indicating adequate balance. No units were discarded during matching [33,34,35]. After automated selection of a control group, univariate comparisons across groups were performed to confirm appropriate matching of confounding variables. Treatment efficacy differences between groups were further investigated using the Kaplan–Meier survival analysis with the log-rank test and multivariate Cox proportional hazard modeling. Ref. [36] Propensity matching and subsequent analyses were performed using the R statistical software version 4 (R Foundation for Statistical Computing, Vienna, Austria). A *p*-value < 0.05 was considered statistically significant. 

## 3. Results

Baseline patient and PAVM characteristics, follow-up, treatment data, and success rates of embolization are presented in Table 2. 

The mean patient age was 44.3 ± 16.5 years (range 10–87 y), and 68.5% (74/108) were female. HHT diagnosis was confirmed in 76.8% of patients (83/108), determined by either genetic testing or fulfillment of at least three out of four Curacao clinical criteria [4]. The lower-lobe location accounted for 60.3% of all PAVMs (*n* = 177). Simple angioarchitecture accounted for 65.0% of PAVMs (*n* = 177), while 35.0% were complex (*n* = 95). The average fluoroscopy time was 48.9 min and was nearly identical between groups, averaging 48.6 min in the non-HVDNF group and 49.7 min in the HVDNF group. The mean overall follow-up or time to recurrence on CTA of the chest was 26.0 ± 28.7 months (0.3–122.4 mo). The non-HVDNF-coil group had significantly longer mean follow-up time compared with the HVDNF-coil group (*p* < 0.0001). The mean feeding-artery diameter was 3.3 ± 1.3 mm (2–11) and not significantly different between groups (*p* = 0.17).

### 3.1. Persistent-Occlusion Rates

The overall persistent-occlusion rate was 86.0% (234/272). Persistent occlusion was achieved in 81.8% of PAVMs using non-HVDNF coils (157/192), compared with 96.3% using HVDNF coils (77/80) (*p* = 0.0017). 

### 3.2. Embolic Quantity and Cost Analysis

The overall mean number of coils per PAVM, regardless of coil type, was 5.2 ± 4.4 coils (range 1–36). Within-group analysis showed that the number of coils required for PAVM occlusion was significantly fewer when using HVDNF coils (mean 3.9 ± 3.6 coils, range 1–23) compared with non-HVDNF coils (mean 5.7 ± 4.5 coils, range 1–36) (*p* < 0.0001).

Cost analysis revealed an average embolic cost of USD 4212 ± 3687 (USD 164–27,937) per PAVM. The use of HVDNF coils was more expensive than standard coils, with an average embolic cost of USD 4775 ± 3553 (1540–20,152) for HVDNF coils compared with USD 3977 ± 3725 (164–27,937) for non-HVDNF coils (*p* = 0.0012). When accounting for PAVM angioarchitecture, there was no statistically significant difference in price between groups when treating complex PAVMs (*p* = 0.82). 

### 3.3. Propensity Matching 

Using logistic-regression-derived propensity scores, PAVMs treated with HVDNF coils (*n* = 80) were matched to non-HVDNF-treated controls (*n* = 80) across a set of clinically relevant covariates (Table 3). Statistical tests of differences of means and proportions demonstrated no significant differences between control and treatment groups after matching [37]. 

**Figure 5:**
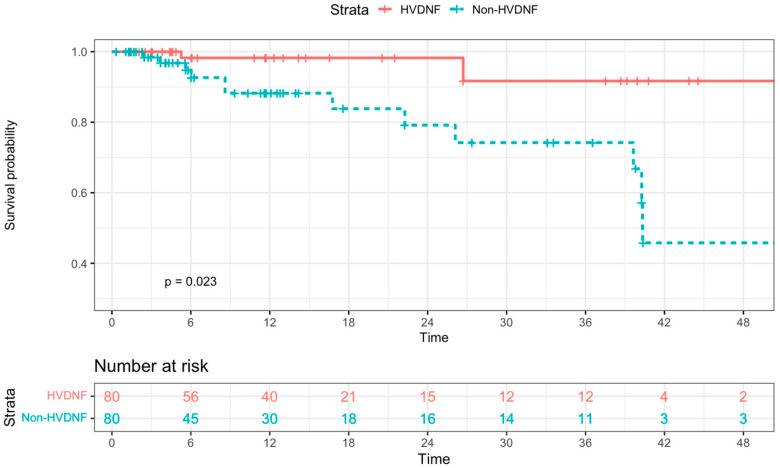
Kaplan–Meier curve of de novo PAVM embolizations, including 80 treated with HVDNF coils and 80 propensity-matched non-HVDNF coils. HVDNF treatment group demonstrated higher recurrence-free survival rate relative to non-HVDNF control group (log-rank test, *p* = 0.023).

The Kaplan–Meier plot demonstrates that PAVMs treated with HVDNF coils recurred significantly less frequently than PAVMs treated with non-HVNDF coils (Figure 5) (log-rank test, *p* = 0.023). After propensity matching, Cox proportional modeling demonstrated increased risk of recurrence with complex angioarchitecture (HR = 3.95, *p* = 0.025) and decreased risk of recurrence with HVDNF coils (HR = 0.19, *p* = 0.021) (Table 4).

## 4. Discussion

Durable transcatheter embolization of de novo PAVMs is dependent on several factors, including angioarchitecture, embolization technique, embolic devices, and time. Persistent-occlusion rates after coil embolization of previously untreated PAVMs differs widely among studies due to different embolization tools and techniques, variable modality, and timing of follow-up [1,3,4,7,8,10,11,12,13,14,15,16,17,18,19,20]. PAVM-embolization device options include fibered pushable coils, detachable fibered coils, hydrogel-coated coils, vascular plugs, and, more recently, high-volume detachable non-fibered (HVDNF) coils (Penumbra Inc., Alameda, CA, USA). HVDNF coils are available in longer lengths and have a larger diameter than comparable fibered microcoils, creating a high-volume embolic. These properties, combined with a soft coil design, are intended to maximize packing density and produce cross-sectional occlusion without relying on thrombus formation. 

To date, there are no convincing data that establish the superiority of newer-generation coil embolics in achieving persistent occlusion. Prasad et al. (2004) retrospectively evaluated the efficacy of platinum versus stainless-steel coils in 306 PAVM embolizations and found no significant difference in persistent-occlusion rates on follow-up CT of the chest (89.7% vs. 93.3%, *p* = 0.5) [14]. Kennedy et al. (2020) evaluated PAVM recurrence rates in a prospective randomized controlled trial of detachable 035 Interlock coils versus pushable 035 Nester coils [20]. The study was prematurely terminated and thus underpowered; however, there was no significant difference in recurrence rates or complications in 20 detachable versus 26 pushable PAVM treatments at the mean 1.1-year follow-up (5.6% vs. 0%, *p* > 0.05). 

There are several investigations that demonstrate vascular plugs alone or in combination with coils may be superior to coil embolization alone [19,22,38,39,40,41,42]. Trerotola et al. (2010) retrospectively reviewed 37 embolizations of PAVMs with 5 mm or larger feeding arteries performed with Amplatzer vascular plugs (AVP, St. Jude Medical, St. Paul, MN, USA) with the addition of fibered coils [22]. There was 100% persistent occlusion at the mean 13-month follow-up. In a larger retrospective study, Bailey et al. (2019) reviewed 119 PAVM embolizations with Microvascular Plugs (MVP, Medtronic, Minneapolis, Minnesota) and also demonstrated a 100% persistent-occlusion rate at the mean 10.8-month follow-up [38]. 

Andersen et al. (2019) retrospectively reviewed the frequency of recurrence and outcomes in 322 PAVM embolizations over a period of 20 years [19]. Embolics included primarily fibered pushable coils (*n* = 213), AVP (*n* = 89), and AVP with coils (*n* = 7). The mean follow-up was 58.0 months, and recurrence rates were 11.7% for coils compared with 4.5% for AVP (*p* = 0.07). Coil types were not separately evaluated.

In the current investigation, the overall treatment success for de novo PAVMs was 86.0%, which is in line with prior studies. The overall persistent-occlusion rate of de novo PAVM coil embolization was higher when HVDNF coils were used. After accounting for differences between the groups by using a propensity-matched analysis, the use of HVDNF coils continued to be associated with a significantly higher persistent-occlusion rate compared with non-HVDNF coils. Complex angioarchitecture and use of non-HVDNF coils were associated with increased risk of PAVM recurrence in our population.

Physicians at our institution selected HVDNF coils more often for complex PAVMs and for PAVMs with larger feeding vessels. These are typically harder PAVMs to treat, making the high persistent-occlusion rate of HVDNF coils more impressive. The average embolic cost was significantly higher using HVDNF coils relative to non-HVDNF coils. However, although the use of HVDNF coils was associated with a significantly higher cost than traditional coils when treating simple PAVMs, the cost was similar between groups for the treatment of complex PAVMs. In these groups, the lower cost of traditional coils was offset by the need for fewer HVDNF coils to achieve technical success.

There are several limitations for this study. This study was retrospective in nature, and the sample size for the HVDNF group was relatively small. There was significant heterogeneity within the subgroups. The mean follow-up time was significantly longer for the non-HVDNF-coil group, which may have overestimated the persistence rates seen in this subset, although time was accounted for within the propensity-matched analysis. Non-HVDNF coils included a number of detachable low-volume coils and hydrogel-coated coils, which are distinct from typical traditional coils. There were three pediatric PAVM embolizations in the non-HVDNF-coil group, which may increase the risk of reperfusion, compared with only one pediatric PAVM embolization in the HVDNF group. Embolization techniques were not specifically evaluated or included in the propensity-matching evaluation. Nearly all embolization procedures were performed by a single operator over the course of a decade, with the majority of high-volume-non-fibered-coil embolizations being performed in the later years, which may have improved outcomes in this group due to increasing operator skill; of note, fluoroscopy times did not significantly differ between groups, although this variable alone does not necessarily reflect operator proficiency. Evaluation of outcomes by computed tomography has limitations, particularly if there are dense coil packs with streak artifacts, and therefore may not always be accurate. Additionally, embolic cost may not be generalizable due to institutional variability. This study did not include a comparison to vascular-plug embolics, which have demonstrated excellent occlusion rates in previous series.

## 5. Conclusions

Coil embolization of de novo PAVMs has a high rate of technical success and durable occlusion. Durable transcatheter embolization is dependent on several factors, including angioarchitecture, embolization technique, embolic devices, and time. The use of high-volume detachable non-fibered coils was associated with higher persistent-occlusion rates when compared with non-HVDNF coils. HVDNF coils were more expensive on average; however, the cost was similar between groups for the treatment of complex PAVMs.

## Figures and Tables

**Table 1:** Coil embolic data.

Embolic Brand	Coil Category	Manufacturer		Total
Interlock	Detachable Fibered	Boston Scientific, Marlborough, Massachusetts, USA		650
		Interlock 018	529	
		Interlock 018 soft	50	
		Interlock 035	71	
Axium	Detachable Non-Fibered	Medtronic, Minneapolis, MN, USA		58
Tornado	Pushable Fibered	Cook Medical, Bloomington, IN, USA		50
Ruby	High-Volume Detachable Non-Fibered (HVDNF)	Penumbra, Alameda, CA, USA		209
		Ruby Standard Coil	61	
		Mandrel diameter, range (3–14 mm)		
		Coil length, range (12–60 cm)		
		Ruby Soft Coil	84	
		Mandrel diameter, range (2–8 mm)		
		Coil length, range (4–60 cm)		
		Packing Coil	62	
		Coil length, range (15–60 cm)		
		POD Coil (POD4, POD8)	2	
Nester	Pushable Fibered	Cook Medical, Bloomington, IN, USA		210
microNester	Pushable Fibered	Cook Medical, Bloomington, IN, USA		132
Azur	Detachable Non-Fibered Hydrogel	Terumo Medical, Somerset, NJ, USA		31
Concerto	Detachable Fibered	Medtronic, Minneapolis, MN, USA		64
				1404

**Table 2:** Treatment groups.

Embolic Group	Age * (Years)	Female *	HHT *	Follow-Up (Months)	Angioarchitecture		Fluoroscopy Time (Minutes)	Feeding-Artery Diameter (mm)	Embolic Quantity	Cost Analysis (Dollars)	Success
					Simple	Complex			Number of Coils		
Group 1: Non-HVDNF Coils (*n* = 192)	43.3 ± 17.1 (10–87)	73.2%	85.4%	30.7 ± 31.9 (0.3–122.4)	67.7% (130/192)	32.3% (62/192)	48.6	3.2 ± 1.2 (2–11)	5.7 ± 4.5 (1–36)	3977 ± 3725 (164–27,937)	81.8% (157/192)
Group 2: HVDNF Coils (*n* = 80)	45.4 ± 15.8 (16–69)	68.4%	73.6%	14.7 ± 13.4 (1.2–56.8)	58.8% (47/80)	41.3% (33/80)	49.7	3.4 ± 1.3 (2–10)	3.9 ± 3.6 (1–23)	4775 ± 3553 (1540–20,152)	96.3% (77/80)
TOTAL	44.3 ± 16.5 (10–87)	68.5%	76.8%	26.0 ± 28.7 (0.3–122.4)	65.0% (177/272)	35.0% (95/272)	48.9	3.3 ± 1.3 (2–11)	5.2 ± 4.4 (1–36)	4212 ± 3687 (164–27,937)	86.0% (234/272)
*p*-Value				*p* < 0.0001			*p* > 0.05	*p* = 0.17	*p* < 0.0001	*p* = 0.0012	*p* = 0.0017

* Values in this category are per patient and not per PAVM (remainder of variables are per PAVM).

**Table 3:** Results of propensity matching. Clinically relevant covariate mean differences between HVDNF and non-HVDNF groups prior to and following matching. Post-matching statistical tests show no significant difference between treatment and control groups.

	All Data	Matched Data	Treatment	Control	
	Std. Mean Difference (*n* = 265)	Std. Mean Difference (*n* = 160)	HVDNF (*n* = 80)	Non-HVDNF (*n* = 80)	*p*-Value
Age (Years)	0.075	0.030	45.8 (15.4)	46.2 (16.9)	0.73
HHT					
Present	0.24	0.035	68 (85.0%)	69 (86.2%)	0.82
Absent	“	“	12 (15.0%)	11 (13.8%)	
FA Diameter (mm)	0.17	0.14	3.4 (1.5)	3.1 (1.1)	0.63
Angioarchitecture					
Complex	0.18	0.0	33 (41.2%)	33 (41.2%)	1.0
Simple	“	“	47 (58.8%)	47 (58.8%)	
Embolization Technique					
DFA	0.34	0.0	33 (41.2%)	33 (41.2%)	1.0
NFA	“	“	47 (58.8%)	47 (58.8%)	
Follow-Up (Months)	1.25	0.06	14.0 (15.6)	14.9 (13.5)	0.13

*p*-Values from chi-square test for categorical variables and Wilcoxon rank sum test for quantitative variables. DFA, distal feeding artery; FA, feeding artery; HHT, hereditary hemorrhagic telangiectasia; NFA, nidus-and-feeding artery.

**Table 4:** Results of Cox proportional hazard model with estimates of relative risk for PAVM recurrence. Use of HVDNF coils decreases risk of recurrence. Complex angioarchitecture increases risk of recurrence.

Predictors	Risk-Ratio Estimates	CI	*p*-Value
Age (Years)	0.99	(0.96–1.03)	0.61
HHT			
Absent	ref		
Present	0.70	(0.13–3.80)	0.68
FA Diameter (mm)	1.4	(0.79–2.45)	0.25
Angioarchitecture			
Simple	ref		
Complex	3.95	(1.19–13.17)	0.025 *
Embolization Technique			
DFA	ref		
NFA	0.4	(0.12–1.37)	0.14
Coil Type			
Non-HVDNF	ref		
HVDNF	0.19	(0.05–0.78)	0.021 *

* *p*-Values < 0.05. DFA, distal feeding artery; FA, feeding artery; HHT, hereditary hemorrhagic telangiectasia; HVDNF, high-volume detachable non-fibered; NFA, nidus-and-feeding artery.

## Data Availability

Data are contained within the article.
